# Presence of a loner strain maintains cooperation and diversity in well-mixed bacterial communities

**DOI:** 10.1098/rspb.2015.2682

**Published:** 2016-01-13

**Authors:** R. F. Inglis, J. M. Biernaskie, A. Gardner, R. Kümmerli

**Affiliations:** 1 Environmental Microbiology, Swiss Federal Institute of Aquatic Science and Technology (Eawag), Überlandstrasse 133, 8600 Dübendorf, Switzerland; 2 Department of Environmental Systems Science, ETH Zurich, Universitätsstrasse 16, 8092 Zürich, Switzerland; 3 Department of Plant Sciences, University of Oxford, South Parks Road, Oxford OX1 3RB, UK; 4 School of Biology, University of St Andrews, Dyers Brae, St Andrews KY16 9TH, UK; 5 Department of Plant and Microbial Biology, University of Zürich, Winterthurerstrasse 190, 8057 Zürich, Switzerland

**Keywords:** experimental evolution, microbial biodiversity, non-transitive competition, rock–paper–scissor dynamics, siderophore, social evolution

## Abstract

Cooperation and diversity abound in nature despite cooperators risking exploitation from defectors and superior competitors displacing weaker ones. Understanding the persistence of cooperation and diversity is therefore a major problem for evolutionary ecology, especially in the context of well-mixed populations, where the potential for exploitation and displacement is greatest. Here, we demonstrate that a ‘loner effect’, described by economic game theorists, can maintain cooperation and diversity in real-world biological settings. We use mathematical models of public-good-producing bacteria to show that the presence of a loner strain, which produces an independent but relatively inefficient good, can lead to rock–paper–scissor dynamics, whereby cooperators outcompete loners, defectors outcompete cooperators and loners outcompete defectors. These model predictions are supported by our observations of evolutionary dynamics in well-mixed experimental communities of the bacterium *Pseudomonas aeruginosa*. We find that the coexistence of cooperators and defectors that produce and exploit, respectively, the iron-scavenging siderophore pyoverdine, is stabilized by the presence of loners with an independent iron-uptake mechanism. Our results establish the loner effect as a simple and general driver of cooperation and diversity in environments that would otherwise favour defection and the erosion of diversity.

## 1. Introduction

Explaining the maintenance of cooperation and diversity are major challenges for evolutionary ecology [1–5]. The ‘survival of the fittest’ means that cooperative behaviour is prone to exploitation from defectors that free-ride on the help of others and suggests that diversity will be eroded as superior forms displace weaker ones. Theoretical work indicates that spatial genetic structure (i.e. genetically related individuals living in close proximity) may promote both cooperation and diversity [5–9], but other studies have shown that it is certainly not sufficient [10,11] and may even be obstructive [12–15]. Moreover, many biological settings, including some natural aquatic environments, are characterized by a lack of significant spatial genetic structure, yet they still maintain cooperative and diverse communities [16,17]. This raises the question of what other processes can explain the high levels of cooperation and diversity observed in nature.

Beyond evolutionary ecology, economic game theorists have proposed a simple mechanism—the ‘loner effect’—that can maintain cooperation and diversity, even in the absence of spatial genetic structure [18]. The idea is that, in environments that would otherwise favour defection, cooperators can coexist with defectors when in the presence of a loner that has an independent lifestyle and is therefore not involved in the others' social interactions. Specifically, this can occur when the loner strategy outcompetes the defector but then creates conditions that favour the cooperator in turn, leading to cyclical, ‘rock–paper–scissor’ dynamics. In economics, the loner has been envisioned as a cognitive strategy, whereby a human subject opts out of a public goods game to accept an alternative pay-off [19]. However, we suggest that this mechanism may apply widely across the whole diversity of life, including to the mindless social traits of microorganisms. The occurrence of loners could therefore explain the maintenance of cooperation and diversity in a wide range of biological settings that lack significant spatial genetic structure.

Here, we test the hypothesis that the loner effect has application beyond economics and specifically to a system of cooperative public goods production in bacteria. We develop a mathematical model to examine how the loner effect can apply to a public goods dilemma faced by siderophore-producing bacteria, and we experimentally determine whether or not the loner effect can maintain siderophore production and diversity in evolving communities of *Pseudomonas aeruginosa*. This bacterium produces pyoverdine, a diffusible siderophore that is released into the extracellular environment where it can solubilize iron for the producer cell and for other cells in the neighbourhood [20,21]. The non-excludability of pyoverdine means that producer cells (i.e. cooperators) can be exploited by mutant cells that benefit from siderophores but do not produce any themselves (i.e. defectors) [22–24]. Furthermore, pyoverdine requires a specific receptor for uptake, and a great diversity of pyoverdine-receptor pairs exists among *P. aeruginosa* and other *Pseudomonas* strains [25,26]. Consequently, in natural *Pseudomonas* communities, cooperator strains likely face both exploitation by defectors and competition from other *Pseudomonas* strains with their own iron-acquisition systems (i.e. loners). This could set the stage for rock–paper–scissor dynamics driven by the succession of cooperators, defectors and loners.

## 2. Material and methods

### (a) A mathematical model of strain dynamics in *Pseudomonas* communities

To predict the dynamics and stability of *Pseudomonas* communities, we developed a mathematical model based on our observations of siderophore biology. In the basic model, we consider a large population of bacterial cells, with a proportion *p*_C_ producing and using costly siderophores (cooperators), a proportion *p*_D_ only using the producer's siderophore (defectors) and the remaining proportion *p*_L_ = 1 − *p*_C_ – *p*_D_ producing and using a different, less-efficient siderophore (loners), in a context where access to siderophores is necessary for growth. We use this basic model to examine a two-strategy system consisting of cooperators and defectors only (*p*_L_ = 0) and the complete three-strategy system consisting of cooperators, defectors and loners. Later, we add an extension to examine the occurrence of de novo mutant strategies.

We assume that cells in the population are well mixed (i.e. no spatial genetic structure), but we allow for social interactions to occur in local neighbourhoods of *n* cells (e.g. as reported for bacterial interactions on suspended particles in an otherwise poorly structured, aquatic environment [27]). Our assumption implies that the timescale of social interaction is fast, and the timescale of the cellular birth–death process is slow, relative to the mixing of cells within the entire population. Accordingly, the composition of a focal cell's neighbourhood is a random draw from the multinomial distribution, with parameters *n* − 1 and *p* = (*p*_C_, *p*_D_, *p*_L_). Note that, while we consider stochasticity at the level of individual cells, in terms of the random composition of their social environments, these effects average out at the level of the whole population, which comprises a very large number of cells. Hence, we assume that the overall population dynamics are deterministic.

We consider that a siderophore-producing cell retains a fraction *γ* of its siderophores (as postulated by Zhang & Rainey [28] and Kümmerli & Ross-Gillespie [29]) and releases the remainder 1−*γ* as a public good. A fraction *α* of the public good is shared equally among all individuals in the neighbourhood who are able to use it (including the producer), and the remainder 1−*α* is shared equally among all individuals in the population who are able to use it. It is important to note that we assume that siderophores are not lost due to diffusion, which is a simplifying but reasonable assumption for closed and well-mixed systems, where siderophores and cells can meet at any point in time and space. Consequently, a cooperator cell enjoys a pay-off *P*_C_ = *γ* + (1 − *γ*)(*α*{[1 + *x*_C_]/[1 + *x*_C_ + *x*_D_]} + (1 − *α*){[*p*_C_]/[*p*_C_ + *p*_D_]}) − *c*, where *x*_C_ and *x*_D_ are the multinomially distributed numbers of (other) cooperators and defectors in its neighbourhood, respectively, and *c* is the cost of siderophore production (scaled so the benefit associated with a unit of the cooperator's siderophore is 1). Accordingly, a defector's pay-off is *P*_D_ = (1 − *γ*)(*α*{[*x*_C_]/[1 + *x*_C_ + *x*_D_]} + (1 − *α*){[*p*_C_]/[*p*_C_ + *p*_D_]}) and a loner's pay-off is *P*_L_ = *b* − *c*, where *c* < *b* < 1 describes the relative efficiency of the loner's siderophore. We also extend the basic model to consider the evolution of a ‘loner-defector’ (LD) strategy, which exploits the loner's siderophore and occurs with frequency *p*_LD_. This yields pay-offs *P*_C_ = *γ* + (1 − *γ*)(*α*{[1 + *x*_C_]/[1 + *x*_C_ + *x*_D_]} + (1−*α*) {[*p*_C_]/[*p*_C_ + *p*_D_]}) − *c*, *P*_D_ = (1−*γ*)(*α*{[*x*_C_]/[1 + *x*_C_ + *x*_D_]}+(1−*α*){[*p*_C_]/[*p*_C_ + *p*_D_]}), *P*_L_ = *γb* + (1−*γ*)(*α*{[1 + *x*_L_]/[1 + *x*_L_ + *x*_LD_]} + (1 − *α*){[*p*_L_]/[*p*_L_ + *p*_LD_]})*b* − *c* and *P*_LD_ = (1 − *γ*)(*α*{[*x*_L_]/[1 + *x*_L_ + *x*_LD_]} + (1 − *α*){[*p*_L_]/[*p*_L_ + *p*_LD_]})*b*.

We derive the Malthusian growth rate *m*_S_ of a strain adopting strategy S ∈ {C, D, L, LD} and use this to calculate each strain's change in frequency over time, d*p*_S_/d*t.* Specifically, the growth rate of strain S is given by 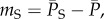 where 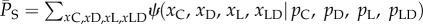 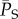 is the average pay-off among all cells of that lineage, 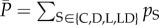 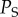 is the average pay-off among all cells in the population, and *ψ*(*x*_C_, *x*_D_, *x*_L_, *x*_LD_|*p*_C_, *p*_D_, *p*_L_, *p*_LD_) = ([*n* − 1]!/[*x*_C_!*x*_D_!*x*_L_!*x*_LD_!])(*p*_C_*^x^*^C^ *p*_D_*^x^*^D^*p*_L_*^x^*^L^*p*_LD_*^x^*^LD^) is the multinomial probability distribution function. In addition to these growth pay-offs, we assume that strain frequencies may change by non-selective processes (including mutation, plasmid infection or migration), occurring at rate *μ*, hereafter described as the ‘mutation rate’. For simplicity, and without affecting the results, we consider that mutations change a particular strain type into the type that can directly outcompete it (i.e. C → D, D → L, L → C or LD) [30]. After incorporating the mutation rate, the deterministic rate of change of each strain is2.1*a*
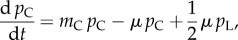
2.1*b*
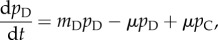
2.1*c*
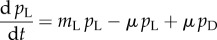
2.1*d*
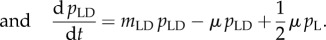
This formulation applies to the four-strategy system, but the three-strategy system (cooperator–defector–loner) or the two-strategy system (cooperator–defector) is recovered by setting *p*_LD_ = 0 or *p*_L_ = *p*_LD_ = 0, respectively, and by changing the mutational connectivity accordingly.

### (b) *Pseudomonas* strains used for empirical tests

We used a total of 11 strains of *P. aeruginosa* (for a complete list of strains, see the electronic supplementary material, table S1). As the cooperator, we used the standard laboratory strain PAO1 (ATCC 15692), a wild-type producer of pyoverdine type 1 and its fluorescently tagged variant PAO1-*gfp* (isopropyl-β-_D_-thiogalactopyranoside (IPTG)-inducible, green-fluorescent marker, chromosomal insertion *att*Tn7::Ptac-*lacI*-e*gfp*). As the defector, we used PAO1Δ*pvdD pchEF* and its fluorescently tagged variant PAO1Δ*pvdD pchEF*-*echerry* (IPTG-inducible, red-fluorescent marker, *att*Tn7::Ptac-*lacI*-*echerry*), knockout mutants that neither produce pyoverdine type 1 nor pyochelin (the secondary siderophore of this species). We used a double-knockout mutant to rule out pleiotropic effects associated with pyoverdine deletions, such as the upregulation of pyochelin in particular. Pyochelin is typically repressed when pyoverdine is produced (as in our cooperator and loner strain) but is synthesized at high rates in pyoverdine-negative strains [31]. By using a double-knockout strain, we could entirely focus on the effect of pyoverdine (since none of the three strains produced pyochelin) and rule out all potential side effects introduced by pyochelin production (for instance, the possibility that the loner and the cooperator exploit the pyochelin produced by the defector). In one instance where it was an appropriate control, we also used PAO1Δ*pvdD*, a strain that is defective for pyoverdine production only. Finally, we considered six putative loner strains, all of which are clinical or environmental isolates, and thus represent natural (rather than engineered) loners that cooperators and defectors might face in real-world settings (electronic supplementary material, table S1). All putative loners produce a pyoverdine type that is different from the one produced by the cooperator PAO1 [25]. We assume that the different pyoverdine types mainly differ in their affinity to iron (i.e. in the benefit they can generate) and not in their production costs because the pyoverdine synthesis machinery is similar in all strains [26].

### (c) Competition assays

We carried out competition experiments by inoculating 7.5 µl of both competing strains at an OD (optical density at 600 nm) of 1 into 1.485 ml of iron-limited media consisting of casamino acids (CAA) medium (5 g casamino acids, 1.18 g K_2_HPO_4_ × 3H_2_O, 0.25 g MgSO_4_ × 7H_2_O l^–1^) and supplemented with 20 mM NaHCO_3_ (sodium bicarbonate) and 100 µg ml^−1^ human apo-transferrin (all from Sigma-Aldrich, Switzerland) in 24-well cell culture plates. Plates were subsequently incubated at 37°C and shaken at 220 r.p.m. for 24 h. Colony forming units (CFUs) were counted by serially diluting and plating each competition on lysogeny broth (LB) agar supplemented with 1 mM FeCl_3_ and 1 mM IPTG. Plates were incubated for 24 h at 37°C and left at room temperature (20°C) for another 48 h to allow the fluorescent markers to mature. A transilluminator (DR88X, Clare Chemical Research) was used to differentiate between the fluorescently tagged strains. The relative fitness of each strain in each competition was calculated as *v* = [*q*_2_(1 − *q*_1_)]/[*q*_1_(1 − *q*_2_)], where *q*_1_ and *q*_2_ are, respectively, the initial and final proportions of the bacterial strain of interest [32].

To first check whether the fluorescent markers affected strain fitness, we competed our marked strains against their unmarked ancestral variants in iron-limited CAA medium. We could not detect any significant difference in relative growth between marked and unmarked strains (relative fitness of marked strain did not differ from *v* = 1, the expected value, for *gfp*-marker: *v* = 1.51 ± 0.63, one-sample *t*-test, *t*_7_ = 1.585, *p* = 0.16; for *mcherry*-marker: *v* = 1.05 ± 0.14, one-sample *t*-test, *t*_7_ = 0.644, *p* = 0.54). This shows that strain dynamics should not be influenced by the fluorescent markers.

Next, we tested whether strains of *P. aeruginosa* producing a different pyoverdine type can act as loners (i.e. can outcompete defectors but get outcompeted by cooperators). To do this, we first performed pairwise competition experiments between the six putative loner strains against PAO1 and PAO1Δ*pvdD pchEF* (four replicates per competition). These assays identified strain ATCC 013 as a potential loner (i.e. outcompeting the defector, but losing against the cooperator). In order to confirm that these competitive differences were in fact due to pyoverdine, and not just based on other competitive factors affecting growth rate, we repeated the competition experiments in CAA medium supplemented with 20 µM FeSO_4_. In this iron-replete environment, the production of pyoverdine is not required, and therefore we expect the loner effect to disappear. In particular, the defector and cooperator strain become phenotypically identical when iron is replete (as none of the strains produces pyoverdine), and consequently the loner should perform similarly against both strains (i.e. either win, lose or grow equally well). Furthermore, we used the strain triplet PAO1, PAO1Δ*pvdD pchEF* and ATCC 013, to test four key assumptions of our model: (i) access to pyoverdine is required for growth; (ii) pyoverdine production is costly (*c* > 0); (iii) the producer cell retains a proportion of its pyoverdine for its own use (*γ* > 0) and (iv) the pyoverdine produced by the loner is less efficient than the pyoverdine produced by the cooperator (*b* < 1). The exact methods for these tests are described in the electronic supplementary material and [33,34].

### (d) Experimental evolution

We used experimental evolution to test our model predictions about strain dynamics and community stability. A primary aim of these experiments was to compare the dynamics and stability of two-strain communities, initially composed of cooperators and defectors only, with that of three-strain communities, initially composed of cooperators, defectors and loners. For this comparison, we performed experimental evolution with replicate communities (*n* = 12) over 10 days (approx. 50 bacterial generations). Separately, we also performed experimental evolution with all two-strain communities (cooperator versus defector, cooperator versus loner, loner versus defector) over 5 days (approx. 25 bacterial generations) in sixfold replication for each strain combination. Experimental evolution was carried out across relatively short timescales because *Pseudomonas* strains are known to rapidly adapt to laboratory conditions, and such adaptations could distort the evolutionary dynamics of interest [35].

Evolution experiments were carried out in 24-well plates, filled with 1.5 ml of iron-limited CAA media, and bacterial culture (50 µl of each strain at the start, OD of each strain adjusted to 1). To prevent contamination between wells, we filled every second well only (hence 12 of the 24 wells were inoculated). Plates were subsequently placed in a 37°C incubator and shaken at 220 r.p.m for 24 h. Following growth, 150 µl of bacterial culture was transferred to fresh media every 24 h for the duration of the experiment. After transferring to new media, the bacterial competitions were serially diluted and plated on iron-supplemented LB agar. These were incubated for 24 h at 37°C and left at room temperature (20°C) for another 48 h. CFUs were counted using a transilluminator.

### (e) Screening of evolved clones

To assess the emergence of de novo defectors in pyoverdine-producing strains (i.e. PAO1 and ATCC 013) in our evolution experiments, we assayed pyoverdine production in these strains over time. In the 10-day experiments comparing two-strain (cooperator–defector) and three-strain (cooperator–defector–loner) communities, we screened pyoverdine producers at days 5 and 10 of the experiment (120 clones for each community). In the 5-day experiments with two-strain communities (cooperator versus defector, cooperator versus loner, loner versus defector), we screened 48 clones from each community for pyoverdine production daily of either PAO1 (in the cooperator versus defector and cooperator versus loner competitions) or ATCC 013 (in the loner versus defector competition). Each bacterial competition was plated on iron-supplemented LB agar, and clones of either PAO1 or ATCC013 were isolated from each replicate. Pyoverdine production was assayed by culturing these clones in a 96-well plate containing 200 µl iron-limited CAA for 24 h at 37°C. Pyoverdine production for each clone was quantified using the RFU/OD measure described above. We defined strains as de novo defectors if their RFU/OD was 75% or less than that of the ancestral wild-type (for further details, see the electronic supplementary material, figure S2).

## 3. Results

### (a) Identification of a putative *Pseudomonas* loner strain

We found that one of the six candidate strains, ATCC 013, behaved as a loner—that is, it was outcompeted by the cooperator (relative fitness with 95% confidence intervals *v* = 1.60 ± 0.18, one-sample *t*-test, *t*_11_ = 6.57, *p* < 0.0001), but outcompeted the defector (*v* = 1.24 ± 0.15, one-sample *t*-test, *t*_11_ = 3.24, *p* = 0.008; electronic supplementary material, figure S1). Crucially, the ATCC 013 loner and the PAO1 cooperator each produce a different, mutually exclusive pyoverdine type [36]. Competition in iron-replete media, where no pyoverdine is produced, confirmed that the above competitive differences were due to the differential production and use of pyoverdine, and not due to other factors affecting strain growth rates. Specifically, the loner effect disappeared in iron-replete media, where ATCC 013 significantly outcompeted the cooperator (*v* = 0.58 ± 0.24, one-sample *t*-test, *t*_7_ = 43.4, *p* = 0.012) and the defector (*v* = 1.62 ± 0.45, one-sample *t*-test, *t*_7_ = 2.7, *p* = 0.031; electronic supplementary material, figure S2). Moreover, as expected given that the competition between cooperators and defectors was dependent on iron availability, the defector outcompeted the cooperator under iron-limited conditions (*v* = 2.6 ± 1.04, one-sample *t*-test, *t*_7_ = 3.03, *p* = 0.019; electronic supplementary material, figures S1 and S2), but not under iron-replete conditions (*v* = 0.8 ± 0.31, one-sample *t*-test, *t*_7_ = 1.25, *p* = 0.25; electronic supplementary material, figure S2), where pyoverdine production is switched off. Altogether, these results suggest that ATCC 013 could act as a true loner in a community of PAO1 (cooperator) and PAO1Δ*pvdD pchEF* (defector) in the iron-limited conditions where pyoverdine is required for growth.

### (b) Testing model assumptions

We used our strain triplet (PAO1, PAO1Δ*pvdD pchEF*, ATCC 013) to test key assumptions of our model, and found all of them to hold: (i) siderophores are important for growth, as indicated by pyoverdine producers growing significantly better than pyoverdine-deficient defectors (cooperator OD after 24 h = 0.601 ± 0.036, loner OD = 0.460 ± 0.007, defector OD = 0.041 ± 0.0001; two-sample *t*-tests: cooperator versus defector, *t*_9_ = 30.8, *p* < 0.0001, loner versus defector, *t*_9_ = 120.9, *p* < 0.0001); (ii) the cost *c* of siderophore production is substantial, as confirmed by our estimate of *c* = 0.197; (iii) the fraction *γ* of siderophores that producers retain for themselves is low, as supported by our iron-chelation assay revealing *γ* = 0.05 and (iv) the cooperator's siderophore is more efficient in delivering iron to the cell than is the loner's siderophore (*b* < 1), as demonstrated by our iron-chelation assays yielding *b* = 0.74 for the loner's pyoverdine.

### (c) Loss of diversity in two-strain *Pseudomonas* communities

In pairwise competition, our model predicts that cooperators will displace loners (given *b* < 1), loners will displace defectors (given *b* > *c*), and—if siderophores are sufficiently costly (large *c*)—defectors will replace cooperators. As an illustrative example of the latter result, we derive analytical results for a simple case with *n* = 2, *γ* = 0 and *α* = 1 (but we emphasize that our model applies more generally). In this case, we find that cooperation is an evolutionarily-stable strategy (ESS; [37]) when siderophores are cheap (*c* < 1/2) and defection is an ESS when siderophores are costly (*c* > 1/2) (see the electronic supplementary material).

**Figure 1.**
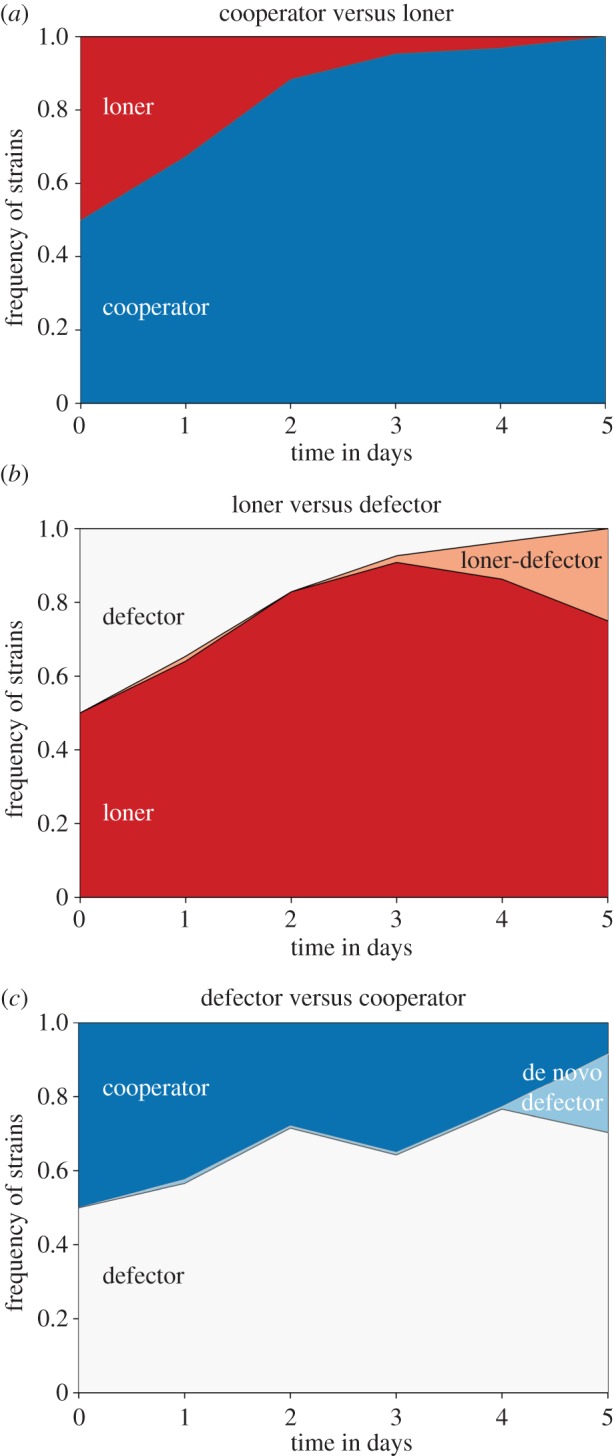
Loss of diversity in two-strain communities and the origin of de novo defectors. Coexistence is not possible in two-strain communities, where (*a*) the cooperator (dark blue) displaced the loner (red); (*b*) the loner (red) displaced the defector (white) and (*c*) the defector (white) outcompeted the cooperator (dark blue). In addition, de novo defectors arose both in the loner and the cooperator backgrounds (salmon-coloured and light blue-coloured branches, respectively).

In support of these predictions, we found that in experimental evolution with two-strain *Pseudomonas* communities: cooperators displaced loners (figure 1*a*, see also the electronic supplementary material figure S3a for individual replicates), loners displaced defectors (figure 1*b* and electronic supplementary material, figure S3*b*), and defectors outcompeted cooperators (figure 1*c* and electronic supplementary material, figure S3*c*). Even though defectors steadily increased in frequency during the initial phase of the experiment when competed against cooperators, we found that by day 5 their increase had slowed and that this coincided with the emergence of de novo defector mutants (i.e. mutants that produce significantly less pyoverdine than the ancestral wild-type) in all replicates (figure 1*c* and electronic supplementary material, figures S4 and S5). The appearance of de novo defectors in the cooperator background is a well-described phenomenon [22,23], and these defectors are known to spread because of an improved siderophore exploitation strategy [38]. In support of this, we found that by the end of our 10-day experiments, a mixture of de novo and ancestral defectors had displaced cooperators in 11 out of 12 replicates (electronic supplementary material, figures S4 and S5). This strongly suggests that, in our well-mixed experimental setting, defectors ultimately displace cooperators.

### (d) Maintenance of cooperation and diversity in three-strain *Pseudomonas* communities

After experimentally confirming that all strain pairs of cooperators, defectors and loners cannot coexist in tandem, we theoretically examined the necessary conditions for all three strains to coexist. Building on our illustrative example above (with *n* = 2, *γ* = 0 and *α* = 1), we find that if individuals may adopt a loner strategy then, while cooperation remains an ESS when siderophores are cheap (*c* < 1/2), there is no pure-strategy ESS when siderophores are costly (*c* > 1/2) and a mixture of all three strategies is maintained. The mixture admits a locally stable equilibrium point at which the pay-offs for the three strategies are equal, but the strain frequencies always cycle around this equilibrium (see the electronic supplementary material). Thus, the presence of the loner promotes both cooperation and diversity through rock–paper–scissors dynamics. This is because the presence of loners in a cooperator's neighbourhood reduces the number of cells that can use the public good, thus increasing the cooperator's share. When cooperation is rare, loners outcompete defectors and rise to high frequency, which alters the social environment in a way that promotes cooperation. A necessary condition for the promotion of cooperation and maintenance of polymorphism is that loners do not ‘soak up’ the cooperator's public good (i.e. take it up without benefiting from it [39]; see the electronic supplementary material for a model variant with soaking).

**Figure 2.**
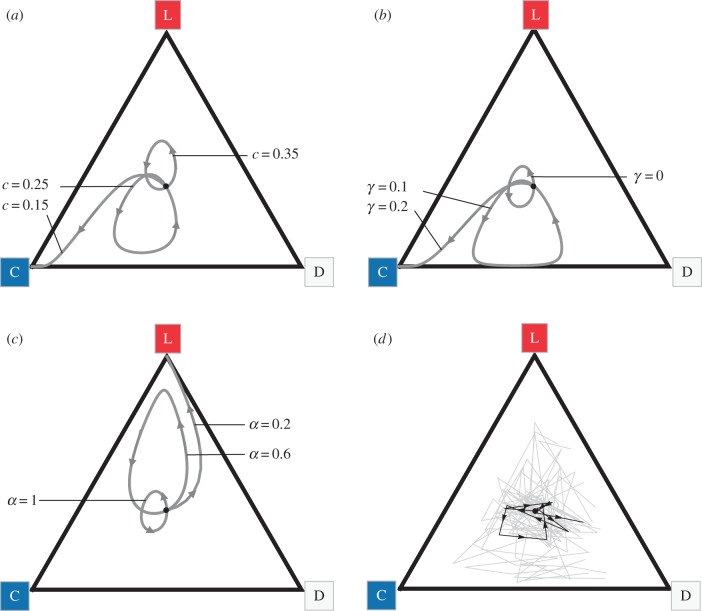
Maintenance of cooperation and diversity in three-strain communities. Triangular plots of deterministic numerical analyses, showing that in communities consisting of a cooperator (C), a defector (D) and a loner (L), rock–paper–scissors dynamics arise when: (*a*) the cost *c* of siderophores is sufficiently high; (*b*) the proportion *γ* of siderophores retained by the cooperator for personal use is low and (*c*) the proportion *α* of siderophores being shared in local neighbourhoods is high. All analyses start with strain types at equal frequency (one-third, shown by the black circle) and, unless otherwise specified, parameters are *c* = 0.3, *b* = 0.7, *α* = 1, *γ* = 0, *n* = 5, *μ* = 0. (*d*) Experiments with three-strain communities of *Pseudomonas* bacteria recover the theoretically predicted rock–paper–scissors dynamics (grey lines show 12 independent replicates; black line shows the mean over 10 days of experimental evolution).

We use a numerical analysis to recover the analytical results above and to confirm that the same qualitative dynamics obtain for more realistic scenarios with *n* > 2, *γ* > 0 and *α* < 1 (figure 2*a–c*; see also the electronic supplementary material, figure S6a–c for time-series plots and figure S7 for additional parameter combinations). Cooperation remains an ESS when siderophores are sufficiently cheap, although larger neighbourhood size tends to inhibit pure cooperation (e.g. when *γ* = 0 and *α* = 1, cooperation is an ESS only if *c* < 1/*n*). This is because, in a larger neighbourhood, a focal cooperator would receive a smaller share of the benefit from its own siderophores. By contrast, when siderophores are sufficiently costly (e.g. *c* > 1/*n* when *γ* = 0 and *α* = 1), all three strategies are maintained in rock–paper–scissors dynamics so long as *γ* is sufficiently low and *α* is sufficiently high (figure 2*a*–*c*), such that most siderophores are shared as public goods within the producer's neighbourhood (see further details in the electronic supplementary material). Larger neighbourhood size tends to increase the amplitude of cycles (results not shown), implying that, in a finite population of large social neighbourhoods, cyclical dynamics could be destroyed by the stochastic fixation of a single type.

Next, we used experimental evolution to directly examine community stability and strain dynamics in communities initially composed of cooperators, defectors and loners. In all replicates, we observed coexistence of all three strains and cyclical shifts in strain frequencies, consistent with the rock–paper–scissors dynamics predicted by our model (figure 2*d*; see also the electronic supplementary material, figure S6*d* for a time-series plot and figure S8 for dynamics of individual replicates). We further examined whether de novo defectors also emerged in the three-strain communities as previously described for our two-strain communities (figure 1). We indeed found that such de novo defectors also evolved in the three-strain communities by day 5 (electronic supplementary material, figures S4 and S5). However, their prevalence was significantly lower than in the two-strain communities (Fisher's exact test for comparisons of de novo defector frequencies after 5 days: *p* = 0.018, and after 10 days: *p* = 0.0001), and the prevalence of the original cooperator strain (producing original pyoverdine levels) was always approximately six-times higher in three-strain communities than it was in two-strain communities (electronic supplementary material, figure S4). These comparisons show that de novo defectors are unlikely to affect community stability in our three-strain communities. However, in addition to these de novo defectors, we also observed the evolution of de novo loner-defectors (electronic supplementary material, figure S3*b*). These are mutants that could potentially exploit the loner's pyoverdine and could therefore undermine community stability in the long term.

### (e) Maintenance of cooperation diversity in four-strain *Pseudomonas* communities

**Figure 3.**
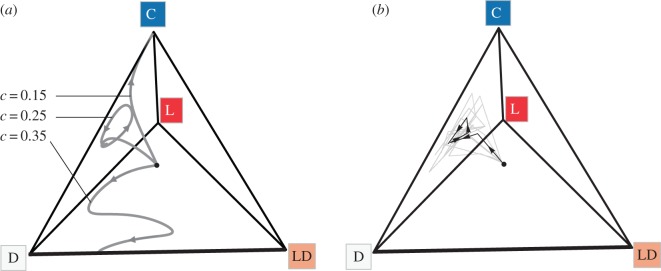
Maintenance of cooperation and diversity in four-strain communities. (*a*) Tetrahedral plot of deterministic numerical analyses, showing that in communities consisting of a cooperator (C), a defector (D), a loner (L) and a de novo evolved loner-defector (LD), C will dominate when the cost *c* of siderophores is low; the two defectors will dominate when *c* is high and LD will go extinct, while the other three strains return to cyclical rock–paper–scissors dynamics, when *c* is intermediate. All analyses start with strain types at equal frequency (one-fourth, shown by the black circle) and, unless otherwise specified, parameters are *b* = 0.7, *α* = 0.8, *γ* = 0.05, *n* = 5, *μ* = 0. (*b*) Experiments with four-strain *Pseudomonas* communities support the intermediate-cost scenario, as indicated by the significant drop in loner-defector frequency and the rock–paper–scissors cycling of the remaining three strains (grey lines show six independent replicates; black line shows the mean over 5 days of experimental evolution).

To theoretically explore the effect of de novo loner-defectors on community stability, we analysed the complete four-strategy system in equation (2.1). Our analytical model results for *n* = 2, *γ* = 0 and *α* = 1 (see the electronic supplementary material) and numerical analyses for *n* > 2, *γ* > 0 and *α* < 1 reveal that the addition of a loner-defector can result in three possible evolutionary outcomes: (i) when siderophores are cheap (low *c*), cooperation is strongly favoured, which eliminates the loner-defector; (ii) when siderophores are costly (high *c*), both defector strategies (D and LD) are favoured and maintained in a stable polymorphism; (iii) when the cost of siderophores is intermediate, the loner-defector is eliminated and the three remaining strains display rock–paper–scissors dynamics (figure 3*a*; see also the electronic supplementary material, figure S9*a* for a time-series plot and figure S10 for additional parameter combinations).

To determine which of these outcomes applies to our experimental system, we isolated six independent de novo loner-defectors at random and competed them against the other three strains in the community (i.e. cooperator, defector and loner). We found that the loner-defectors outcompeted the ancestral loner (relative fitness with 95% confidence intervals *v* = 1.65 ± 0.09, one-sample *t*-test, *t*_5_ = 14.74, *p* < 0.0001). By contrast, we could detect no fitness advantage enjoyed by the loner-defectors over the defector (*v* = 1.70 ± 1.61, one-sample *t*-test, *t*_8_ = 0.82, *p* = 0.42), and all loner-defectors dramatically lost out in competition with the cooperator (*v* = 0.10 ± 0.04, *t*_11_ = 48.68, *p* < 0.0001). Given this highly imbalanced fitness network, where loner-defectors are almost completely displaced by cooperators within 24 h, we predicted that the four-strain system will typically be unstable [40]. Indeed, in an evolutionary experiment with four-strain communities, the loner-defector rapidly dropped to low frequency, going extinct in three of the six replicates, while the original three strains displayed typical rock–paper–scissor dynamics (figure 3*b* and see also the electronic supplementary material, figure S9*b* for a time-series plot and figure S11 for dynamics of individual replicates). Hence, the emergence of loner-defectors does not necessarily eliminate the cooperation and diversity that is maintained by the presence of a loner.

## 4. Discussion

Our results yield novel insights into how cooperation and diversity can be stabilized in real-world natural communities. Building on previous theory [18], our models of public-goods-producing bacteria predict that cooperator and defector strains can coexist with a loner strain, using its own independent public good, in cyclical rock–paper–scissor dynamics (figure 2*a*–*c*). This prediction was supported by our observations of open-ended evolutionary dynamics in siderophore-producing communities of *Pseudomonas*. Under well-mixed conditions that favour defectors over cooperators (figures 1*c* and electronic supplementary material, figure S5*a*), we found that the presence of a loner strain can stabilize the coexistence of defectors and cooperators (figure 2*d* and electronic supplementary material, figure S8). Moreover, this dynamic coexistence of cooperators, defectors and loners was not destroyed by the origin of defectors that exploit the loner (figure 3 and electronic supplementary material, figure S11). Hence, our results suggest that the loner effect is a robust mechanism that can drive the maintenance of cooperation and diversity in an environment that would otherwise favour defection and the erosion of diversity.

Our model predicts that the loner effect can operate only when public goods sharing is sufficiently local. That is, although we assumed that cells are mixed such that genetically related individuals are not in close proximity (i.e. no spatial genetic structure), the maintenance of cooperation and diversity requires that cells interact in local neighbourhoods [18]. This is because, in the absence of spatial genetic structure, selection can favour public goods production only if the benefit of producing the good sufficiently feeds back to the producer cell itself (a direct fitness benefit of cooperation). Under these conditions, the loner effect works because loners occupy space between cooperators and defectors, thereby shielding the public good from defectors. Specifically, the presence of the loner reduces the number of neighbours that can benefit from the cooperator's public good, which in turn increases the direct fitness benefit that a cooperator cell gains from its own secreted molecules. Hence, our observation that cooperator, defector and loner strains can coexist in *Pseudomonas* communities suggests that, despite interacting in shaken media, there was some mechanism to keep pyoverdine molecules sufficiently local, resulting in a direct benefit of cooperation in the presence of loners. For instance, high cell density could ensure that pyoverdine molecules never diffuse very far before meeting another cell, such that iron-loaded molecules are usually shared within the local neighbourhood of the producer. Alternatively, it has been shown that turbulences, which could have been induced by our shaking procedure, can lead to the formation of micro-scale patches where neighbours, and perhaps their secreted products, stay together [41]. Further studies will be necessary to elucidate these mechanisms and to determine whether they also occur in other well-mixed natural settings.

Our study provides a unique example of how rock–paper–scissor dynamics can maintain biological diversity. Our finding that cooperation and diversity are maintained in well-mixed populations sets our study apart from most previous theoretical and empirical work, where rock–paper–scissor dynamics exclusively occurred in genetically structured environments [42–45]. For instance, Kerr *et al.* [44] showed that coexistence between toxin-producing, toxin-sensitive and toxin-resistant bacterial strains was only possible in environments with both local toxin interactions and local cell dispersal. This is because the selective advantage enjoyed by a rare toxin-producing strain derives from eliminating unrelated cells that are competing locally with their genetic relatives; by contrast, those genetic relatives are locally absent in well-mixed populations, such that the rare toxin-producing strain would enjoy no selective advantage [46]. More recently, Kelsic *et al*. [47] extended the classic antibiotic producer–resistant–sensitive model to include extracellular antibiotic degradation, showing that coexistence of all three-strain types can occur even when cells are well mixed. Hence, similar to the loner effect, this model illustrates that diversity can be maintained when social interactions are local but cell dispersal is global.

Although we have focused on *Pseudomonas* communities in our study, it is important to note that the role of the loner is potentially very general and may even be enacted by organisms belonging to a different taxonomic group than the cooperator and the defector. In aquatic microbial systems, for example, the loner could be a distinct bacterial species or potentially even a small eukaryote, such as an alga. Moreover, our study suggests that the loner strategy is not limited to cognitively advanced economic agents like humans [19] but can also be employed by simple unicellular organisms such as bacteria or amoeba [48]. This highlights that community stability and the maintenance of cooperation are emergent properties of the loner presence and do not require adaptations from the organisms themselves. The loner effect is therefore different from adaptive responses, such as the evolution of kin recognition mechanisms [49]; metabolic optimizations that reduce the cost of public good production [50,51] and the evolution of less-diffusible metabolites [52], which can also contribute to the maintenance of cooperation in the absence of spatial genetic structure.

Finally, we propose that public goods cooperation itself can favour the evolution of loners and may therefore promote diversity via a multi-step evolutionary process. This is in fact evidenced by our open-ended evolutionary study in which bacteria were not confined to an artificially minimal strategy set but were instead able to innovate new strategies in a naturalistic way. Similarly, we expect that in a well-mixed population of cooperators, defectors will first arise and enjoy an evolutionary advantage. In turn, these defectors may modify their environment in a way that favours the evolution of loners whose public goods are accessible to neither cooperators nor defectors. The resulting three-strain community can be either: (i) transitive, and reverting to a one-strategy community dominated by a superior loner; (ii) non-transitive, and leading to rock–paper–scissors dynamics, as observed in our study or (iii) open-ended, and evolving into higher-order systems with multiple cooperators and defectors coexisting, at least temporarily [40]. Our results provide an empirical basis to theoretical work having proposed that social interactions mediated by toxins [53], signalling molecules [54] and siderophores [30] can drive diversification in microbial communities [55]. Our study of the loner effect adds a simple and general principle to a number of alternative mechanisms—including density reduction via interspecific competition [56] and shared antibiotic degradation [47]—that may be common in well-mixed microbial communities and could contribute to maintenance of both cooperation and biodiversity.

## Data accessibility

Data available from the Dryad Digital Repository: http://doi.org/10.5061/dryad.k5n4k.
